# Long‐Ranging Movements of Asiatic Lions: Implications for Conservation and Management in Gujarat, India

**DOI:** 10.1002/ece3.71811

**Published:** 2025-07-16

**Authors:** Mohan Ram, Aradhana Sahu, Nityanand Srivastava, Rohit Chaudhary, Lahar Jhala, Yashpal Zala

**Affiliations:** ^1^ Wildlife Division Sasan‐Gir Junagadh Gujarat India; ^2^ Wildlife Circle Junagadh Gujarat India; ^3^ Chief Wildlife Warden, Gujarat State Gandhinagar Gujarat India; ^4^ Department of Wildlife Sciences Banda University of Agriculture and Technology Banda Uttar Pradesh India

**Keywords:** Asiatic lion, conservation, cropland, forest, habitat selection, shared landscape

## Abstract

Effective conservation and management of large carnivores in landscapes shared with humans require a comprehensive understanding of their habitat selection and movement patterns. The Asiatic lion ( *Panthera leo persica* ), an endangered species, has experienced population growth and expansion beyond the Gir Forest. This study investigates the factors influencing the habitat selection and movement patterns of lions undertaking long‐distance movements in shared landscapes, providing insights for their long‐term conservation and management. We tracked the movement patterns and habitat selection of 10 Asiatic lions using satellite collars. Movement metrics, including distance traveled, were analyzed across different habitats during the day and night. Space use was evaluated by assessing the time lions spent in various habitats, with core area preferences analyzed using the 50% fixed kernel and Jacob's preference index. Fine‐scale habitat use during day and night was analyzed using the integrated step selection function. Lions moved significantly more distance at night (11.07 ± 0.19 km) than during the day (2.28 ± 0.05 km). In core areas of their home ranges, they showed a strong preference towards orchards, followed by forests and water bodies, while avoiding built‐up areas and cropland. The integrated step‐selection function indicated that habitat selection was positively associated with natural habitats such as forests, wastelands, and water bodies (day: 3.31 ± 0.32; night: 0.50 ± 0.12). Conversely, built‐up areas negatively impacted habitat use (day: −0.45 ± 0.41; night: −0.93 ± 0.14), with orchards (day: 2.83 ± 0.33; night: −0.14 ± 0.13) and cropland (day: 1.42 ± 0.32; night: −0.86 ± 0.12) showing variable selection between day and night. Lions in shared landscapes traveled greater distances during the night, likely to avoid human disturbances. Natural habitats—forests, wastelands, and water bodies—are crucial for their space use and habitat selection, underscoring the importance of these areas for their long‐term conservation and management.

## Graphical abstract

This study examines the habitat selection and movement patterns of Asiatic lions in shared landscapes, tracking 10 lions using satellite collars. Results show lions travel more at night, likely to avoid human disturbances, preferring natural habitats like forests, orchards, and water bodies, while avoiding human‐built areas. The findings emphasize the importance of natural habitats for long‐term lion conservation and management.

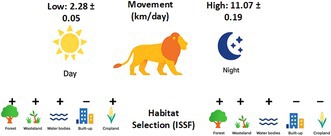

## 1 Introduction

The expansion of human activities has led to the loss and fragmentation of wildlife habitats worldwide (Doherty et al. 2021). Approximately 50%–70% of the Earth's land surface has been altered for human use (Sterling and Ducharne 2008). These changes in landscape composition and configuration have profoundly impacted various ecological processes, including wildlife movements. Recent studies (Tucker et al. 2018) have demonstrated that terrestrial mammals exhibit reduced activity in areas with high human presence compared to those with low human activity. This negative impact on movement is especially pronounced for large carnivores, which have large spatial requirements. Due to their high energy needs and large body size, these species are significantly affected by linear infrastructure, such as roads and railways (Tucker et al. 2018). Restricted movement in large carnivores disrupts their social structures, alters their dietary habits, and affects predator–prey dynamics (Tucker et al. 2018; Valeix et al. 2012; Habib et al. 2021). Additionally, when large carnivores move through shared landscapes, they often prey on domestic livestock, leading to conflicts with local communities. To cope with human disturbances, these carnivores may shift their activities to nocturnal hours, which can have ecological and physiological consequences (Levy et al. 2019). Therefore, understanding the factors that influence large carnivores' movement and habitat selection in human‐modified landscapes is crucial for long‐term conservation and management.

Advances in radio tracking technology and devices have made it possible to collect detailed data on wildlife movement and space use, offering valuable insights for conservation strategies (Lahoz‐Monfort and Magrath 2021). These technological advances are complemented by analytical techniques such as integrated step selection functions and hidden Markov models (McClintock and Michelot 2018; Signer et al. 2019), which help identify factors affecting habitat selection and movement patterns, as well as different states of wildlife movement, such as resting and traveling. Data from GPS collars, which provide location information at regular intervals, can address questions related to wildlife movement and habitat ecology, aiding in conservation and management planning in shared landscapes.

The Asiatic lion ( *Panthera leo persica* , hereafter referred to as lion), found in the Saurashtra region (20°50′–23°5′ N and 69°20′–72°10′ E) of Gujarat, India, has experienced population growth over the past decades (Ram, Vasavada, Tikadar, Jhala, and Zala 2023). Historically, Asiatic lions ranged from Turkey to eastern India. However, their habitat and population suffered significant declines due to hunting and habitat loss. Intensive conservation efforts, first initiated by the Nawab of Junagadh and later sustained by the government of Gujarat, have successfully preserved and increased their numbers. As of the latest population estimate conducted in June 2020, the core population comprises 344 lions, with an additional 330 lions distributed across seven satellite populations within the Saurashtra region (Ram, Vasavada, Tikadar, Jhala, and Zala 2023). Since 2015, the lion population has demonstrated an average annual growth rate of 5.77% (Ram, Vasavada, Tikadar, Jhala, and Zala 2023). This increase is attributed to factors such as stringent protection measures (Singh and Gibson 2011), an increase in native wild prey populations (Ram, Sahu, Srivastava, Chaudhary, Jhala, and Zala 2023), available habitats within the Gir and Greater Gir landscape (Singh and Gibson 2011), the establishment of corridor linkages to the Gir forest (Meena et al. 2014; Vasavada et al. 2022), religious reverence, and local support for lion conservation (Vasavada et al. 2022; Meena et al. 2021). The Gir forest is surrounded by a multi‐use landscape that includes agricultural, horticultural, and plantation crops, coastal areas, industrial zones, and built‐up areas (areas dominated by physical structures such as residential and commercial) (Ram, Vasavada, Tikadar, Jhala, Zala, and Meena 2023; Ram et al. 2024), as well as lion habitats in the form of forest patches, scrubland (dense, thorny shrub‐dominated habitat with scattered trees and open patches, providing cover and hunting opportunities), wasteland (open, sparsely vegetated land, often degraded or barren, but used by lions for movement and resting), and areas around water bodies like rivers and dams (Ram et al. 2022). Furthermore, mixed‐use landscapes create distinct ecological conditions compared to protected areas, which in turn influence lion dispersal and movement strategies.

As the lion population in the Gir forest has grown, individuals have dispersed into the surrounding multi‐use landscape, where they face challenges such as high human presence, linear infrastructures, and comparatively low availability of wild prey. The limitations of optimal habitats and territories in the surrounding landscape compel lions to move long distances in search of suitable habitats and territories (Ram, Vasavada, Tikadar, Jhala, Zala, and Meena 2023). During these long‐distance movements, lions are more likely to encounter anthropogenic pressures compared to those with established home ranges, which exhibit site fidelity and frequently revisit known areas. Previous studies (Ram, Vasavada, Tikadar, Jhala, Zala, and Meena 2023) focused on lions with established home ranges, demonstrating movement patterns characterized by repeated use of areas. Latter study found that forests, along with wastelands, serve as critical habitats for lion conservation. In contrast, some lions did not maintain settled territories for extended periods and were primarily roamers throughout the study. These individuals exhibited erratic movements, settling only briefly for very short periods. Due to this behavior, they were excluded from previous studies. However, their sporadic and unpredictable movements offer an opportunity to gain new insights into habitat selection and movement patterns for an increasing population of lions expanding into human dominated landscapes.

**FIGURE 1:**
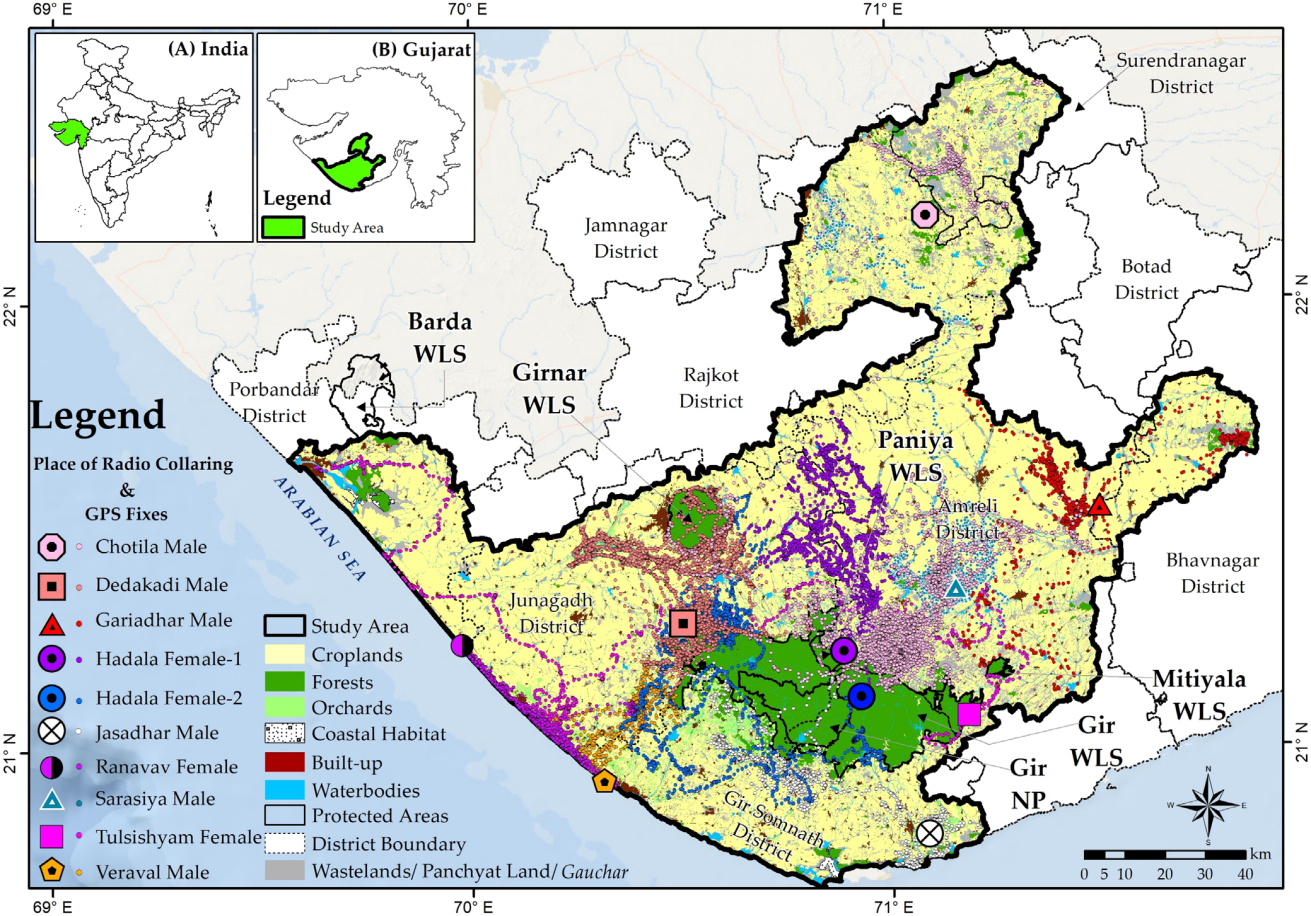
Study area map indicating the locations where 10 Asiatic lions were radio‐collared and their subsequent location fixes. Insets show the position of Gujarat state (A) within India and the study area (B) within Gujarat state.

In our study area, cropland was the dominant land use type (69.32%), followed by wasteland (13.63%), forests (8.01%), water bodies (3.30%), built‐up areas (3.14%), and orchards (1.47%). This diverse landscape prompted us to focus on long‐ranging lions to understand how varying land use impacts their ecology, thereby informing conservation and management efforts. Thus, this study was conducted in the shared landscape of Saurashtra (Figure 1) to understand the spatial and movement ecology of long‐ranging lions. The objectives were: (1) to assess the movement metrics of long‐ranging lions, (2) to identify factors affecting their habitat selection, and (3) to provide conservation and management implications based on the findings.

Previous studies on large carnivores, including Asiatic and African lions, have found that habitat selection is influenced by the time of day (Jhala et al. 2009; Suraci et al. 2019; Nisi et al. 2022). During the day, lions may avoid built‐up areas or modified habitats (such as cropland). Therefore, we hypothesized that in a shared landscape, lions would avoid built‐up areas and modified habitats like cropland and orchards during the day, while preferring natural habitats such as forests, wastelands, and areas around water bodies. We further hypothesized that during the night, when human disturbances are reduced, lions might exhibit a true preference for modified habitats and built‐up areas. This preference may be driven not only by lower levels of human activity but also by the availability of feral cattle as a supplementary food source.

## 2 Materials and Methods

### 2.1 Study Area

The lions inhabit the Gir and Greater Gir Landscape, collectively known as the Asiatic Lion Landscape. This landscape covers approximately 30,000 km^2^ (Ram, Vasavada, Tikadar, Jhala, and Zala 2023). The landscape spans nine districts in Gujarat, western India, and includes five protected areas: Gir National Park, Gir Wildlife Sanctuary, Paniya Wildlife Sanctuary, Mitiyala Wildlife Sanctuary, and Girnar Wildlife Sanctuary, along with other natural forest patches. These forest patches fall under different legal categories, including reserved forests (RF), protected forests (PF), and unclassed forests (UF) (Vasavada et al. 2022). These forest patches support various prey species, such as spotted deer ( *Axis axis* ), sambar ( *Rusa unicolor* ), and wild pig ( *Sus scrofa* ), in forested habitats. The grasslands and scrublands are home to antelope species like Indian gazelle ( *Gazella bennettii* ), blackbuck ( *Antilope cervicapra* ), and nilgai ( *Boselaphus tragocamelus* ) (Ram, Sahu, Srivastava, Chaudhary, Jhala, and Zala 2023). Cropland in the shared landscapes also supports the nilgai and wild pig populations, key prey species for lions in such areas (Ram, Sahu, Srivastava, Chaudhary, Jhala, and Zala 2023; Ram, Sahu, Srivastava, Chaudhary, and Jhala 2023). Built‐up areas within the multi‐use landscape lack natural prey species but are marked by the presence of free‐ranging cattle, which serve as an important prey source for lions in the landscape (Ram et al. 2022).

The landscape is located in a semi‐arid biogeographical zone (Rodgers and Panwar 1988), with an aridity index ranging from 20% to 40% (Jadav 2010). The region receives an average annual rainfall of approximately 600 mm, primarily during the southwest monsoon (Farooqui et al. 2013). However, the Gir forest has recorded an average annual rainfall of 976.50 mm over the past 25 years (1996–2020) (Vasavada et al. 2022). The mean maximum and minimum temperatures are 34°C and 19°C, respectively (Gundalia and Dholakia 2013). The area experiences three main seasons: summer (March–June), monsoon (July–October), and winter (November–February). The landscape is dominated by agro‐pastoral systems and features diverse natural ecosystems, including thorn scrub forests, grasslands, dry deciduous and riverine forests, mangroves, intertidal regions, and coastal areas, including estuaries (Ram, Sahu, Srivastava, Chaudhary, Jhala, and Zala 2023; Vasavada et al. 2022).

### 2.2 Capture, Selection, Radio‐Collaring, Release, and Monitoring

We deployed Vertex Plus GPS radio collars (Vectronic Aerospace GmbH, Berlin, Germany) on 10 lions, including six males and four females, ensuring that each individual belonged to a different pride. We also ensured the collar weight remained below 3% of the lion's body weight, regardless of age or sex (Table S1). For immobilization, we used a combination of ketamine hydrochloride (2.2 mg/kg body weight; Ketamine, Biowet Puławy sp. z o.o, Poland) and xylazine hydrochloride (1.1 mg/kg body weight; Xylaxil, Brilliant Bio Pharma Pvt. Ltd., Telangana, India), administered using a gas‐powered Telinject G.U.T 50 dart delivery system (Telinject Inc., Dudenhofen, Germany). Standard body measurements were recorded for each immobilized lion following the Gujarat Forest Department's standard operating procedures (SOP). After the collars were deployed, we administered Yohimbine hydrochloride (0.1–0.15 mg/kg body weight; Yohimbe, Equimed, Allentown, NJ, USA) intravenously to reverse the sedation, allowing for full recovery within 5–10 min (Sontakke et al. 2009). Experienced veterinarians and wildlife healthcare teams performed all radio‐collaring procedures.

The study was conducted between June 2019 and December 2023 as part of the Asiatic Lion Radio‐Telemetry Project. Data from the radio‐collared individuals were collected and monitored through the Gir Hi‐Tech Monitoring Unit, a technology‐driven scientific monitoring initiative based in Sasan‐Gir, Gujarat. Each collar had a mortality sensor, a programmable drop‐off feature, and a GPS schedule recording the lion's location every hour, both during day and night.

Understanding the movement behaviors and habitat preferences of these long‐distance roaming lions, especially through advanced analytical techniques and accounting for the time of day, is crucial for effective conservation and management. This study advances our understanding by employing integrated step selection functions to analyze habitat preferences during both day and night, providing a more comprehensive perspective on lion behavior and habitat use.

### 2.3 Data Analysis

#### 2.3.1 Movement Metrics

We examined six habitat variables to analyze movement metrics and habitat selection in relation to the time of day (day/night). The habitat variables included three natural habitats (forest, wasteland, water bodies), two human‐modified habitats (cropland, orchards), and one risky habitat (built‐up areas). In our study, we defined “risky habitats” as built‐up areas, as they pose significant direct and indirect threats to lions through activities such as conflict with humans and increased exposure to anthropogenic hazards. In contrast, “human‐modified habitats” are defined as agricultural areas (here as croplands and orchards), which, while altered by human activity, present a different set of challenges and opportunities for lions, such as altered prey availability and movement patterns. We obtained a land‐use and land‐cover map of the study area from the Bhaskaracharya Institute for Space Applications and Geo‐Informatics, Gandhinagar, Gujarat (BISAG‐N) for the year 2020. The land‐use and land‐cover data were then reclassified and merged into broader categories to align with the Area of Interest (AOI). The forest habitat includes patches of grasslands, scrubs, and thickets of *Prosopis juliflora* and *Casuarina equisetifolia* (in coastal areas), which are crucial for lion conservation and management as they provide refuge amid human disturbances (Vasavada et al. 2022). These habitats also support wild prey, such as spotted deer, nilgai, wild pig, and sambar (in some areas only), which are important components of the lions' dietary requirements (Ram, Sahu, Srivastava, Chaudhary, Jhala, and Zala 2023; Ram, Sahu, Srivastava, Chaudhary, and Jhala 2023). Wasteland and scrubland offer resting sites during the day when forest habitats are not available. Water bodies, including rivers, wetlands, and reservoirs, are considered habitat covariates because the surrounding riparian areas have dense cover, serving as resting and movement areas for lions (Meena et al. 2014).

Cropland consists of crops like sugarcane ( *Saccharum officinarum* ), cotton ( *Gossypium herbaceum* ), millets, soybean ( *Glycine max* ), and groundnut ( *Arachis hypogaea* ). Orchards, mainly cash crops like mango ( *Mangifera indica* ), provide daytime refuges for lions due to their good canopy. Due to high human disturbance and activities, built‐up areas are perceived as risky areas for lions.

We measured lion movement by summarizing the average distance traveled (km ± SE) during the day (07:00 h to 19:00 h, Indian Standard Time) and night hours (19:01 h to 06:59 h, Indian Standard Time) using ArcMap (version 10.8.1, ESRI, Redland, USA). We compared these movements between day and night using a two‐sample *t*‐test (Zar 2006). Additionally, we analyzed the time spent in each habitat type by calculating the average hours lions spent in each habitat during the day and night, with differences assessed via *t*‐tests. All analyses were conducted in R using the “sp,” “raster,” “dplyr,” “geosphere,” and “lubridate” packages in R (R Core Team 2023).

#### 2.3.2 Space Use and Habitat Selection

We assessed space use and habitat selection using two approaches. First, we calculated the core areas for each individual using the 50% fixed kernel (FK) method with the *adehabitatHR* package in R. We selected the 50% Fixed Kernel (FK) as it effectively delineates the core areas of activity, providing insights into the spaces actively utilized and prioritized by lions. We used one location for the day and one for the night for each lion, as opposed to using all the locations in the analysis to avoid the temporal correlation in the locations. This metric is particularly valuable for identifying key zones of concentrated activity, making it essential for understanding habitat selection. We extracted the percentage of different habitat types within each lion's core area and represented these graphically. Habitat use within the core areas was tested for significant differences using the Kruskal–Wallis one‐way ANOVA (Zar 2006). Habitat preference was evaluated using Jacob's index (Jacobs 1974) to compare the percentage of core area usage by lions with the percentage of habitat availability in the study area. Jacob's index ranges from −1 to +1, where values near −1 indicate habitat avoidance and values near +1 indicate preference (Valeix et al. 2012).

In the second approach, we assessed fine‐scale habitat use by employing integrated step‐selection functions (iSSF) from the “*amt*” package in R (Signer et al. 2019; Nisi et al. 2022). The iSSF approach, and the conditional logistic regression upon which it is based, inherently accounts for individual variation by conditioning on the strata defined by each observed step and its associated alternative steps. By focusing on within‐individual comparisons of used versus available steps, the iSSF framework effectively controls for differences between individuals without explicitly including random effects. The iSSF approach also allows for the simultaneous estimation of both habitat selection and movement metrics, such as step length and turn angle, by relaxing the assumption that these two factors are independent (Signer et al. 2019).

The iSSF approach compares the actual movement steps (i.e., the movements between consecutive GPS locations) with a set of randomly generated steps, representing alternative paths the animal could have taken within the movement trajectory. We considered 20 available points for each step using random step lengths and turning angles projected from the lion's previous location. The choice of 20 points was based on earlier studies on African lions, particularly the approach used by Nisi et al. (2022). Given the ecological similarities between African and Asiatic lions, we adopted this species‐specific methodology to ensure consistency in our analysis. These random step lengths were drawn from gamma distributions, whereas the turning angles were drawn from Von Mises distributions fitted to the empirical movement data (Avgar et al. 2016). For this analysis, day and night categorizations were determined using actual solar times, calculated with the ‘Suncalc’ package. Habitat selection was evaluated by extracting habitat variables at the end of each movement step. By comparing the characteristics of these real and random available steps, we assessed the lions' habitat preferences based on utilized habitats.

#### 2.3.3 Model Formation and Evaluation

We constructed five models to assess lion habitat selection based on habitat characteristics (natural, human‐modified, and risky habitats). The first model included all habitat variables and was treated as the null model. The other four models were designed to explore different combinations of habitat types: one with only natural habitats, one with only human‐modified habitats, one combining risky and natural habitats, and one combining risky and human‐modified habitats (Table 1). We used lion movement, represented by step lengths, as a response variable to these models.

**TABLE 1:** Model details for habitat selection by lions.

Model no.	Details	AIC	∆AIC	Model weightage
1	**Null model** Forest+Wasteland+Water bodies+ Built‐up+Cropland+Orchards	156,690.2	0.00	1.00
2	**Risky + Modified Habitat model** Built‐up+Cropland+Orchards	157,596.7	612.57	0.00
3	**Natural Habitat Model** Forest+Wasteland+Water bodies	157,409.6	719.40	0.00
4	**Risky + Natural Habitat Model** Forest+Wasteland+Water bodies+Built‐up	157,302.8	906.49	0.00
5	**Modified Habitat model** Cropland+Orchards	159,541.0	2850.77	0.00

We used the Akaike Information Criterion (AIC) to determine the best‐fitting model. Models with a ΔAIC value of less than 2 were considered the best models for explaining habitat selection (Anderson 2008). The beta coefficients for each habitat covariate were used to evaluate the magnitude and direction of their influence on lion habitat selection, providing insights into which habitats were preferred or avoided. We visualized the beta coefficients using the *ggplot2* package in R.

## 3 Results

### 3.1 Movement Metrics

**FIGURE 2:**
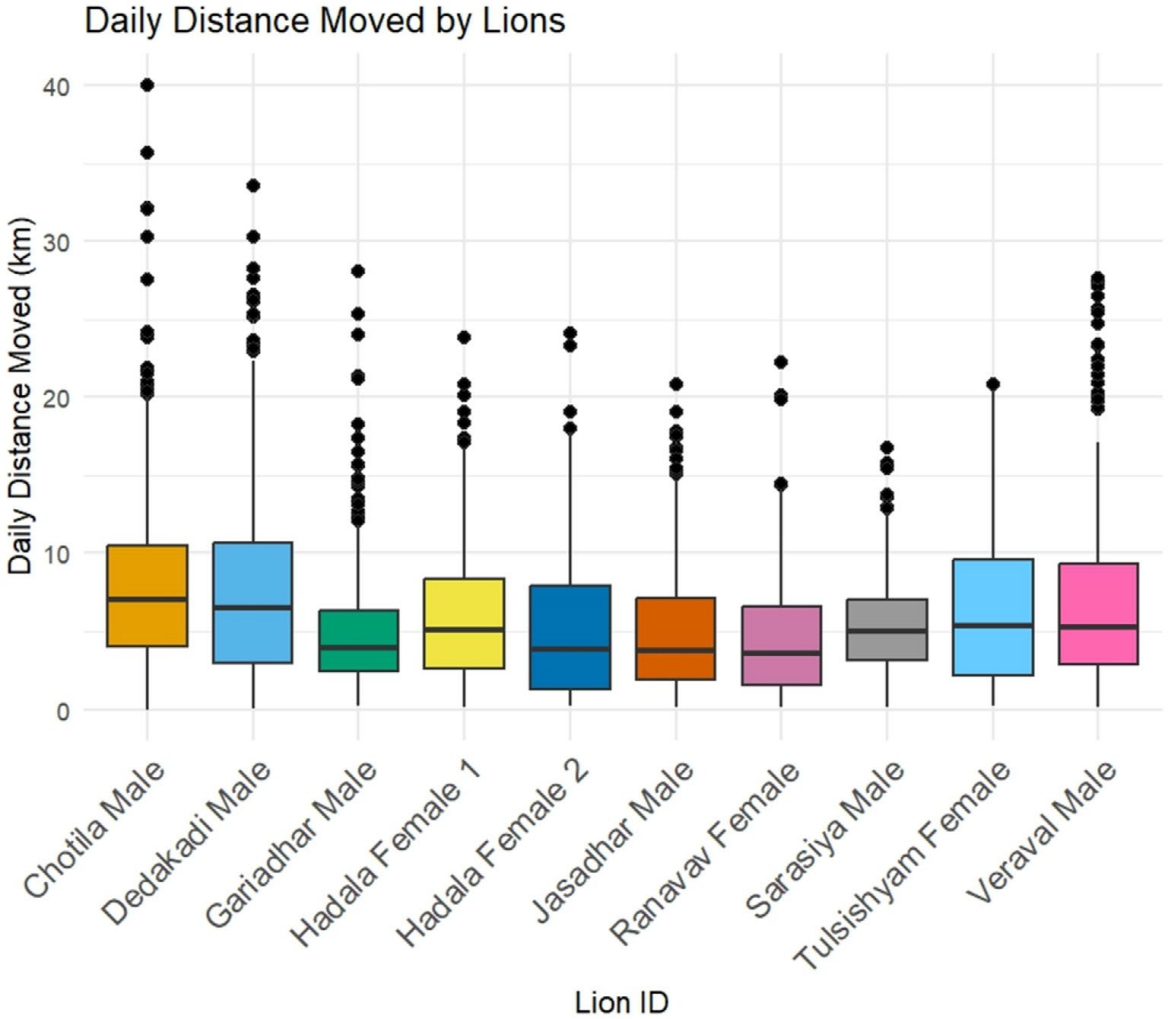
Summary of daily distance moved (in kilometers) by radio‐collared Asiatic lions.

Our dataset, after sorting for regular intervals of 1 h, includes 128,056 location points from 10 lions (Figure 1), among which 62,917 consist of day locations and 65,139 consist of night locations. Over the monitoring period, the lions traveled a cumulative distance of 30,842 km, averaging 3084 ± 748 km per individual. The mean daily movement distance was 6.68 ± 0.12 km (Figure 2).

Lions traveled significantly shorter distances during the day (2.28 ± 0.05 km) compared to the night (11.07 ± 0.19 km) (*t* = 42.51, *p* = 0.001).

**FIGURE 3:**
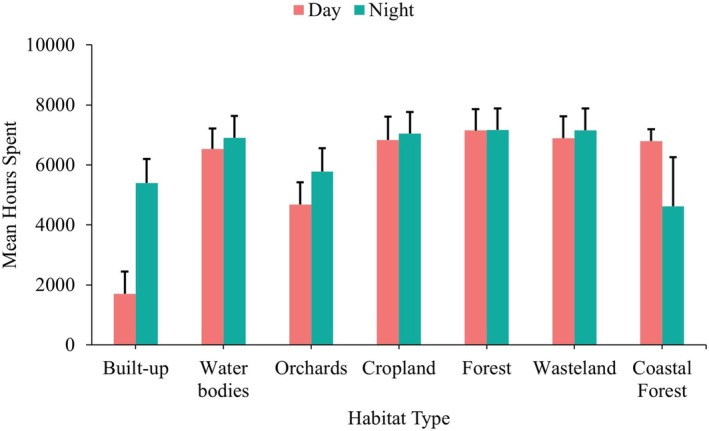
Graph illustrating the time spent (in hours) by lions in different habitat types during day and night hours.

Lions spent the most time (mean) in forests during both day and night, and the least time in built‐up areas (Figure 3). However, there was a significant increase in time spent in built‐up areas at night compared to during the day (*t* = 3.20, *p* = 0.002).

### 3.2 Space Use

The average core area size for lions was 384.24 ± 174.12 km^2^, ranging from 44.61 to 1921 km^2^ (Figures S1–S10; Table S1). Agriculture was the dominant habitat type in the core areas of their home ranges, followed by forests and wastelands, with built‐up areas contributing the least (Figure 4).

**FIGURE 4:**
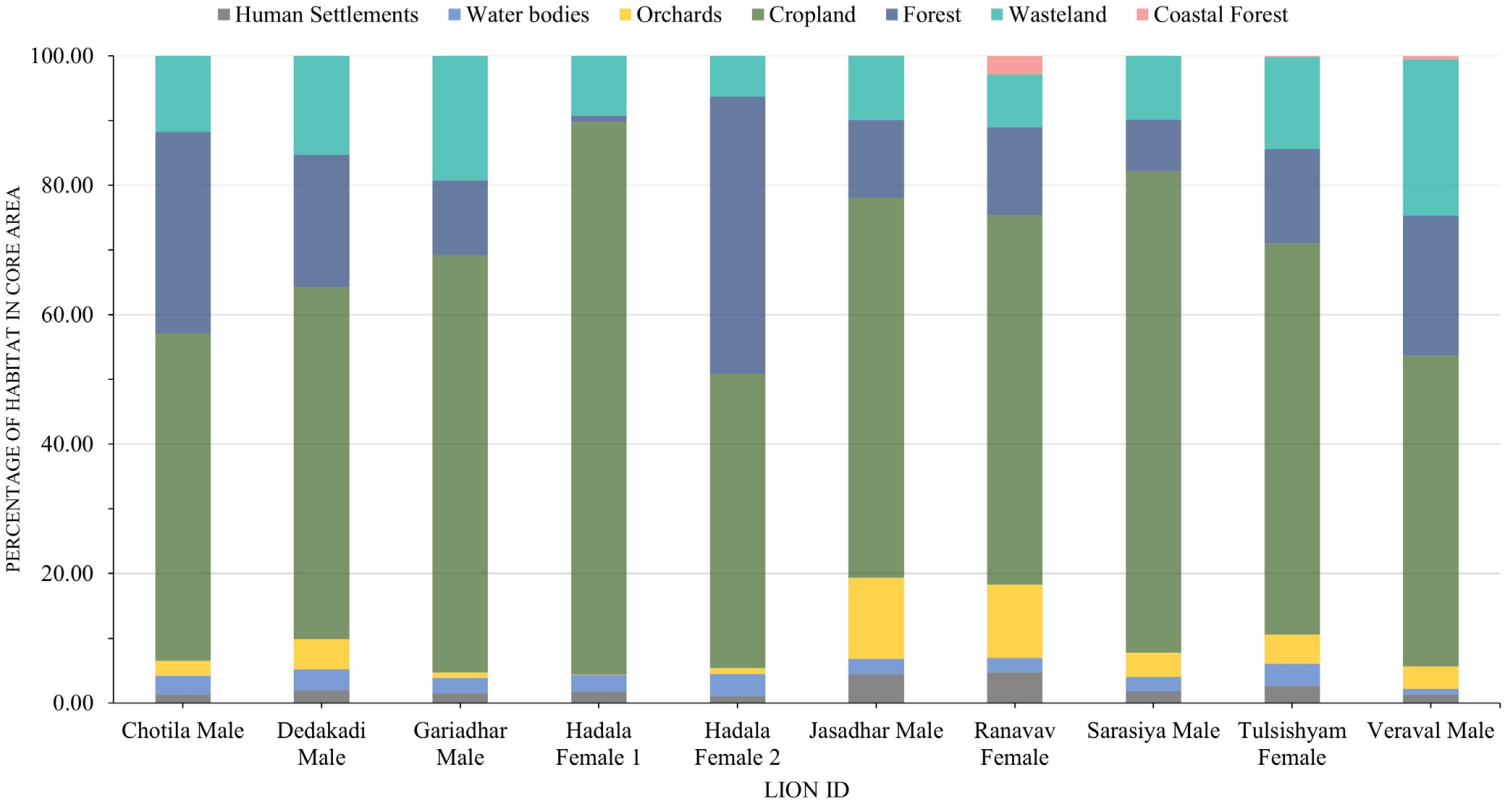
Graph showing the percentage of habitat use by lions across various land‐use classes in core areas of their home ranges.

**FIGURE 5:**
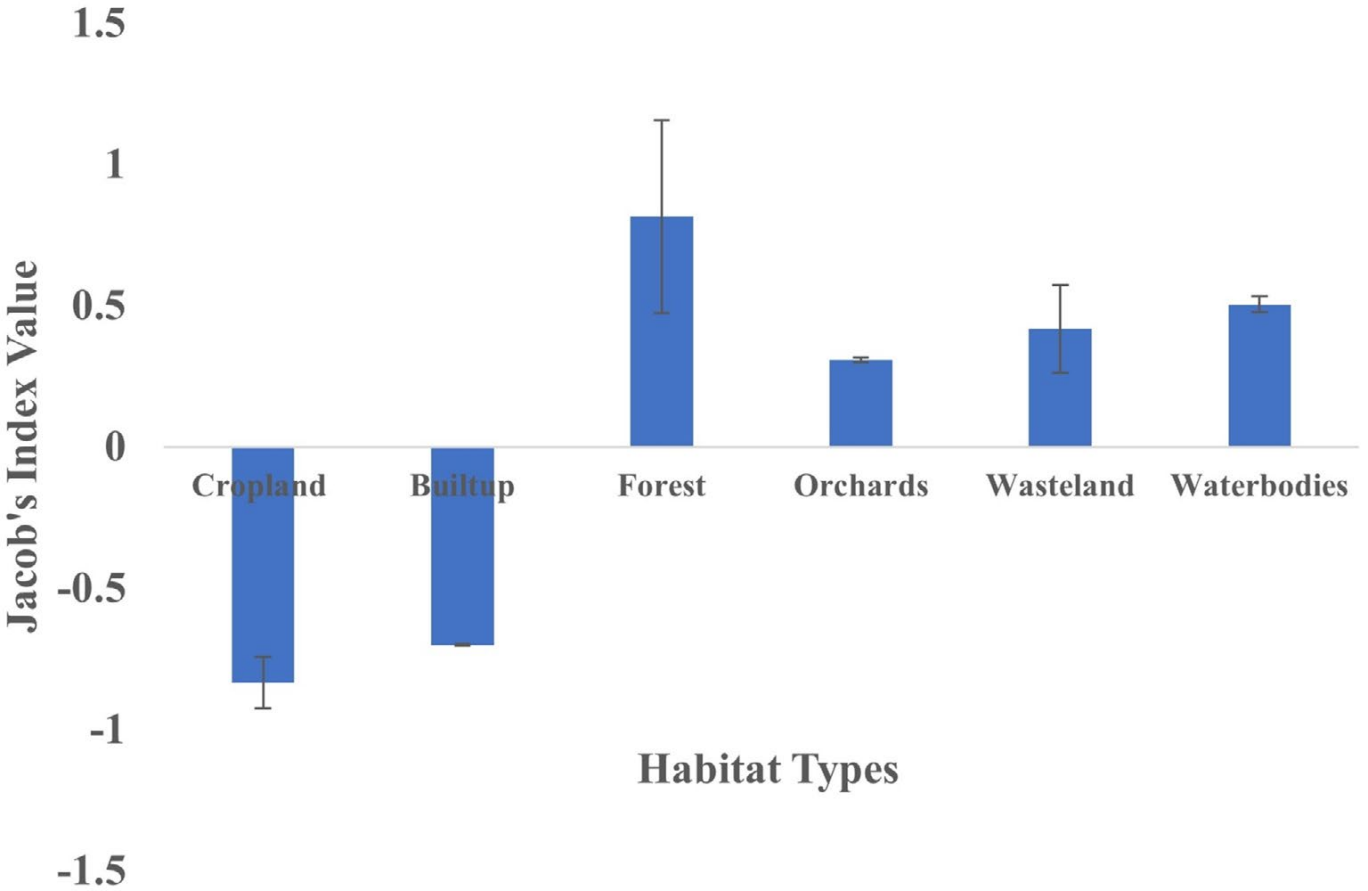
Graphical representation depicting habitat preferences of lions in the core areas of their home ranges. (+ values represent preference, −ve avoidance while 0.00 used in proportion and the bars depicts the standard errors).

There was individual variation in habitat use within core areas of their home ranges. For example, Hadala Female‐1's core area consisted of 85% cropland habitat, whereas Hadala Female‐2's core area contained only 45% cropland. The Kruskal–Wallis test revealed significant differences in habitat use within the core areas of lions (*H* = 55.36; *p* < 0.001). Forests and cropland emerged as important habitats, accounting for over 70% of core area usage for all lions. Long‐ranging lions exhibited a strong preference for forest, followed by water bodies, wasteland and orchards, while they avoided built‐up areas and cropland in core area habitat usage (Figure 5).

### 3.3 Habitat Selection

Among the five models evaluated, the null model (including all habitat variables) was the best‐supported model (ΔAIC = 0) (Table 1).

**FIGURE 6:**
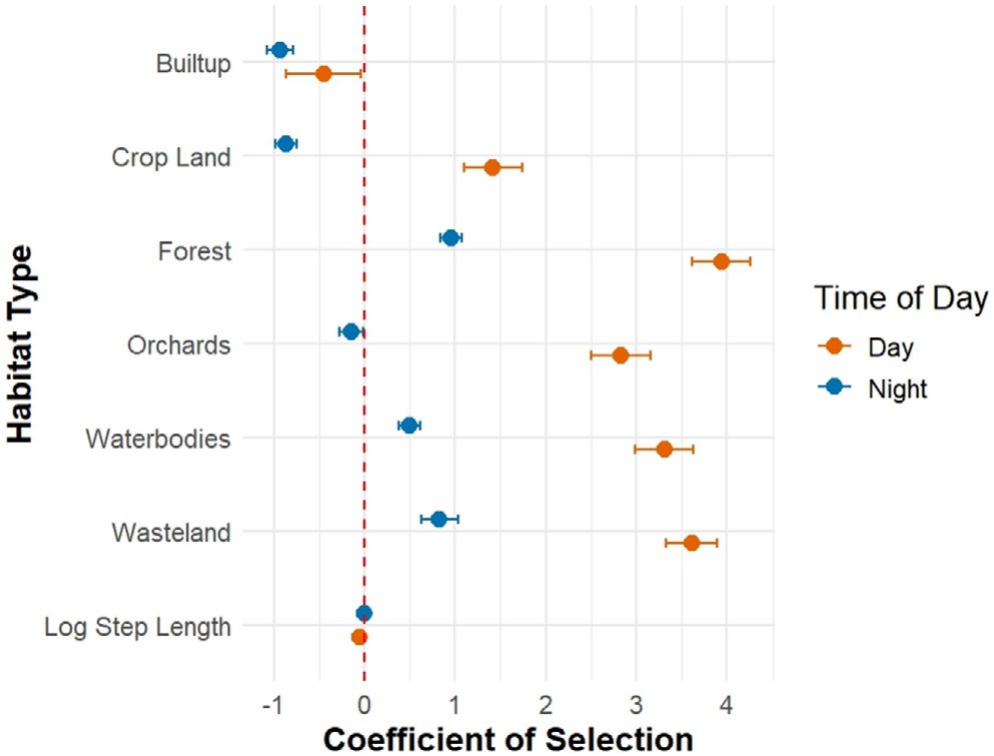
Beta coefficients for different habitat types selected by lions. Positive values denote preference, while negative values indicate avoidance.

During the day, habitat selection was positively influenced by forests, wastelands, orchards, cropland, and water bodies, with built‐up areas having a negative influence. The strongest positive effect was attributed to forests (*β* ± SE: 3.94 ± 0.32), followed by wastelands (3.61 ± 0.28), water bodies (3.31 ± 0.32), orchards (2.83 ± 0.33), and cropland (1.42 ± 0.32). Built‐up areas had the weakest effect, which was also negative (−0.45 ± 0.41) (Figure 6).

At night, habitat use was positively influenced by forests, water bodies, and wastelands, while built‐up areas, cropland, and orchards negatively influenced habitat use. Among the positive influences, forests had the strongest effect (0.96 ± 0.12), followed by wastelands (0.83 ± 0.20) and water bodies (0.50 ± 0.12). For negative influences, built‐up areas had the most substantial negative effect (−0.93 ± 0.14), followed by cropland (−0.86 ± 0.12) and orchards (−0.14 ± 0.13).

## 4 Discussion

Understanding the influence of ecological and anthropogenic factors is fundamental for fostering coexistence between humans and large carnivores in shared landscapes (Nisi et al. 2022).

Our findings revealed that lions traveled greater distances during the night compared to daytime, consistent with previous studies on large carnivores (Habib et al. 2021; Nisi et al. 2022; Naha et al. 2021). Increased human activity during the day likely increases risks for lions, resulting in reduced movement to avoid human encounters. Additionally, high daytime temperatures in the semi‐arid landscape impose energy constraints, leading to less movement distances during the day.

Our study focused on long‐ranging Asiatic lions, which had not established permanent home ranges, allowing us to compare their movement ecology with those of territorial lions previously studied by Ram, Vasavada, Tikadar, Jhala, Zala, and Meena (2023). This comparison provides insights into how different movement strategies influence habitat use and the conservation approaches required for each group. Ram, Vasavada, Tikadar, Jhala, Zala, and Meena (2023) documented the movement and habitat selection of lions with well‐established home ranges, primarily residing within core forested areas and maintaining stable territories. These individuals exhibited predictable movement patterns, largely confined to protected areas or multi‐use landscapes with consistent resource availability. In contrast, our study found that long‐ranging lions traveled significantly greater distances, often venturing beyond protected areas into highly human‐modified landscapes, indicating a different set of ecological and behavioral adaptations.

A key distinction between these groups was their habitat selection. Lions in Ram, Vasavada, Tikadar, Jhala, Zala, and Meena (2023) predominantly occupied forests and adjacent scrublands, avoiding anthropogenic landscapes such as cropland and built‐up areas. In contrast, our results showed that long‐ranging lions exhibited a more opportunistic strategy, frequently utilizing orchards, cropland, and wastelands for movement and temporary refuge. This suggests that long‐ranging lions are more adaptable in utilizing human‐modified habitats, likely due to the necessity of locating resources during dispersal or territorial establishment. Additionally, their higher nocturnal movement rates indicate a behavioral shift to avoid human disturbance, a pattern not as pronounced in the territorial lions studied by Ram, Vasavada, Tikadar, Jhala, Zala, and Meena (2023).

The average time lions spent in different habitats did not show significant variation between day and night, except in built‐up areas. Due to high levels of human activity and limited refuge options, lions used these areas less during the day. However, at night, the time spent in built‐up areas more than doubled. Similarly, Valeix et al. (2012) found that African lions in Botswana's Makgadikgadi ecosystem adapted by hunting livestock at night, when human activity was lower. In the Asiatic Lion Landscape, built‐up areas contain many free‐ranging livestock, which are an important part of the lions' diet outside protected areas (Ram, Sahu, Srivastava, Chaudhary, and Jhala 2023). The increased time lions spend in these areas at night is likely due to their search for free‐ranging livestock, hunting, and feeding during these hours. In contrast, the relatively consistent time spent in other habitat types likely reflects their use as resting places during the day and as movement corridors at night.

The analysis of core area habitat preferences in lions' ranging areas shows a strong preference for orchards, forests, and water bodies, whereas cropland and built‐up areas were avoided. Wastelands were used in proportion to their availability. Core area usage in large carnivores is typically associated with critical resources such as water, prey, and cover, which help meet their needs and minimize human disturbances (Lesilau et al. 2021). In the Asiatic Lion Landscape, lions' preference for natural habitats like forests and water bodies is likely due to the availability of prey and cover, making these habitats key to their core area usage. Interestingly, despite being a human‐modified habitat, lions strongly prefer orchards within their core home range areas. Orchards provide thick canopy cover and water sources and are often well‐guarded, offering good resting sites during the day. These essential resources make orchards highly favored by lions within their core home ranges.

Our hypothesis on habitat selection was supported, as lions exhibited a preference for forests, wastelands, and water bodies during both day and night. Previous studies (Jadav 2010; Schuette et al. 2013; Vanbianchi et al. 2017; McManus et al. 2021) have shown that lions, like other felids such as leopards, pumas, and lynxes, prefer natural habitats like forests, shrublands, and barren lands due to their ecological advantages. Two key factors driving this preference are the availability of refuge and prey (Suraci et al. 2019; Kushata et al. 2018; Sargent et al. 2022).

Forested habitats provide dense cover, making them ideal for resting and cub rearing, particularly in areas with high human activity. They also offer strong protection, minimizing human disturbance (Ram, Sahu, Srivastava, Chaudhary, Jhala, and Zala 2023). Wastelands, though offering less cover, remain important due to lower human pressure and prey availability. In the Asiatic Lion Landscape, these habitats support a diverse prey base, including nilgai, wild pigs, and spotted deer, while croplands and orchards offer limited wild prey (Ram, Sahu, Srivastava, Chaudhary, Jhala, and Zala 2023). Additionally, dense vegetation in forests enhances ambush success, improving hunting efficiency (Hopcraft et al. 2005). Interestingly, lions' habitat preference appears less pronounced at night, likely due to their increased movement through other habitat types when human disturbances are lower.

Water bodies played a significant role in habitat selection during the daytime compared to nighttime. This daytime preference is likely due to two primary factors: the dense vegetation around water bodies, providing cover, and the availability of water to meet lions' physiological needs. The habitat surrounding these water sources supports their requirements for hunting, resting, and movement (Cozzi et al. 2013; De Boer et al. 2010). Additionally, these areas offer thermal cover, stay cooler during the day, and serve as good resting sites. Studies have also shown that riverine habitats can act as natural movement corridors (Lee and Smyth 2003), which may further explain why lions prefer areas near water bodies, as these zones facilitate discreet movement with minimal risk of human detection.

The coastal areas are characterized by extensive stretches *of* *Prosopis juliflora* thickets, *Casuarina* plantations, and diverse coastal vegetation, including sporadic patches of mangroves, rocky and sandy beaches, intertidal mudflats, salt pans, and salt‐affected areas. These habitats support three satellite lion populations distributed along the coast (Ram, Vasavada, Tikadar, Jhala, and Zala 2023; Ram et al. 2022). In addition to various land‐use patterns, the coastal region includes approximately 110 km^2^ of forested areas (Ram et al. 2022). However, the limited use of coastal habitats observed in this study may be due to the small sample size (*n* = 4) of lions that utilized these areas for movement.

Our hypothesis regarding anthropogenic and modified habitats was partially supported, as lions showed a positive association with orchards and croplands during the day but avoided them at night. Previous studies have reported both positive and negative effects of cropland on lion habitat selection (Sargent et al. 2022). In landscapes where natural habitats such as forests and wastelands are scarce, human‐modified habitats like croplands can serve as alternative habitats for large carnivores (Sargent et al. 2022). For instance, Athreya et al. (2013) documented high densities of leopards in agriculture‐dominated areas, whereas Warrier et al. (2020) found that tigers used croplands for cover.

Croplands account for most of the study area, making it difficult for lions, particularly dispersing or long‐ranging individuals, to rely solely on natural habitats for daytime cover. The limited availability of natural habitats might drive the preference for croplands during the day. Additionally, local tree planting practices, such as *Azadirachta indica* , *Syzygium cumini* (along the streams), *Melia azedarach* , *Ficus* species, etc., along cropland edges (Patel and Singh 1996), may favor lions' presence in these areas. Many croplands also feature runnels (small stream or narrow water channel, often referring to tiny rivulets or drainage pathways along the edge of cropland) and seasonal streams along their edges, which stay cool due to water flow seasonally and are relatively less disturbed. These areas are often lined with shrubs, trees, grasses, climbers, and other vegetation, offering vegetation cover and cool, shaded spots for resting. Further, many of these runnels and seasonal streams are enhanced by soil and moisture conservation structures, such as check dams and check dam‐cum‐causeways, which increase the chance of using these areas as resting spots. These features provide cool, shaded areas with moisture, making them attractive resting places for lions during the daytime. This could help explain the positive selection of croplands during daylight hours.

Moreover, croplands with moderate ground cover, as opposed to thick ground cover, support nilgai and feral cattle populations, which comprise over 60% of the lion's diet in this landscape (Ram, Sahu, Srivastava, Chaudhary, and Jhala 2023). Since both nilgai and feral cattle are diurnal, croplands may offer lions greater prey availability during the day (especially morning and evening), with substantial biomass. At night, lions likely avoid croplands due to the reduced activity of their prey, such as nilgai, which are primarily active during the day, and the presence of farmers protecting their crops.

Orchards in our study area are predominantly composed of mango plantations. While Ferreira et al. (2018) highlighted that felids frequently use tree plantations in human‐modified landscapes due to the availability of prey and refuge, this dynamic does not fully apply to our context. In our study area, mango plantations are heavily guarded, especially during the cropping season, limiting prey availability. However, during the noncropping season, these orchards offer lions refuge with reduced human disturbances and cooler microclimates provided by the good canopy cover, particularly during the day. This likely explains the lions' increased use of orchards during daylight hours. At night, however, with reduced human presence and cooler ambient temperatures in other areas, the benefits of using these orchards are diminished, leading to their relatively lower use during nighttime.

We observed that lions' step length decreased during the day, indicating shorter steps and slower movement. This likely reflects a strategy to minimize human encounters and disturbances. At night, with reduced human activity and the advantage of darkness, lions take longer strides and move more quickly. This increase in step length and speed at night may also be associated with activities such as patrolling their territories and searching for prey and mates across larger areas.

Although our study provides valuable insights into lion movement patterns, future research should explore the influence of seasonality on habitat use and movement behavior. Given that many human‐modified landscapes, such as orchards and croplands, undergo seasonal changes in resource availability, it is crucial to investigate how lions adjust their space‐use patterns in response to these fluctuations. Additionally, understanding seasonal variations in prey availability and human activity could further elucidate the adaptive strategies employed by lions. Long‐term monitoring incorporating seasonal dynamics would help refine conservation strategies, ensuring that both territorial and dispersing lions are effectively managed across different times of the year.

## 5 Conservation Implications

The Saurashtra landscape is undergoing rapid development, characterized by increased human settlements, infrastructure expansion, and industrial growth. This evolving environment underscores the need for conservation strategies that harmonize with ongoing development while protecting lion habitats.

Forested areas are essential for extensive lion movement and survival, making it crucial to strengthen efforts aimed at safeguarding these habitats. Priority may be given to habitat restoration, including grassland management and coastal ecosystem preservation. Wastelands, the second most important habitat for lions after forests, are under increasing pressure from agricultural expansion, encroachment, large renewable energy projects, and industrial activities. Some legal protections, such as designating key areas as conservation or community reserves, could help mitigate further habitat fragmentation and loss.

Agroforestry, which incorporates tree cover into croplands and along seasonal streams, offers some refuge and resting areas for lions. Existing government schemes may be leveraged to encourage farmers to adopt such practices. Moreover, strengthening existing government‐supported, beneficiary‐oriented schemes such as *Vany Prani Mitras* (Friends of Wildlife), *machans* (elevated platforms to keep watch in agricultural fields), securing open wells with parapet walls, and eco‐development initiatives in peripheral villages can significantly contribute to the long‐term conservation and management of lions in the landscape. Additionally, encouraging cropland areas within important wildlife corridors to adopt these schemes and grow lion‐friendly crops can benefit both wildlife and farmers. This approach would promote safe movement for lions while reducing human‐lion conflicts. Orchards, which provide significant daytime resting sites for lions, require further study to determine which types and ages of orchards are most beneficial for lion conservation. Such research would help refine strategies to maximize the role of orchards in supporting lion habitat use.

While lions might have adapted to modified habitats such as orchards and croplands in the absence of natural habitats, preserving natural habitats—forests, wastelands, and water bodies—should remain the central focus for long‐term conservation and management. Ensuring habitat connectivity, engaging local communities in conservation efforts, promoting sustainable land‐use practices, and restoring degraded habitats are important for balancing lion conservation with the developmental pressures in the Saurashtra landscape.

The findings of this study underscore the remarkable adaptability of Asiatic lions to human‐modified landscapes, including orchards, which they often use as resting sites during the day. This adaptability highlights the potential for coexistence between lions and humans, mainly when supported by cultural values and traditions of tolerance, as observed among local communities in Saurashtra. Orchard owners could implement management practices that harmonize land use with conservation objectives while minimizing conflicts. Promoting community engagement, as mentioned above, fostering coexistence initiatives, and emphasizing the cultural values of tolerance are critical steps in achieving this balance. These strategies not only ensure the long‐term coexistence of lions and humans but also provide a valuable model for similar conservation efforts in other shared landscapes.

These suggestions align with the “Project Lion” (Gujarat Forest Department 2022), a 25‐year project aimed at comprehensive biodiversity conservation and management in the Saurashtra landscape. It may help ensure lions' long‐term survival in a landscape increasingly influenced by development.

## 6 Conclusion

Understanding large carnivores' movement patterns and habitat use is crucial for long‐term conservation in shared landscapes. Our study reveals that lions adapt their behavior to mitigate the risks of anthropogenic disturbances by predominantly moving at night. They show a strong preference for natural habitats, such as forests and wastelands, which offer slower movement and lower energy expenditure compared to modified and built‐up environments.

In their home range, lions use forests and water bodies extensively in their core areas, emphasizing such areas' importance as lion habitat. Orchards, despite being modified habitats, are also notably used within their core areas, indicating that modified habitats can be valuable if they meet the basic requirements of large carnivores.

Detailed analysis using the integrated Step Selection Function (iSSF) revealed that lions have distinct habitat preferences throughout the day. Natural habitats—forests, wastelands, and water bodies—are crucial both during the day and night, providing refuge and prey. Equally, human‐modified habitats such as orchards and croplands are primarily used for cover during the day and are less utilized at night. Built‐up areas, due to their inherent risks, are avoided consistently throughout the day and night, suggesting these areas may be perceived as unsafe. By addressing these habitat preferences and associated risks, the prospects for long‐term conservation of lions in the shared landscape of Saurashtra may be significantly improved.

## Author Contributions

**Mohan Ram:** conceptualization (equal), data curation (equal), funding acquisition (equal), investigation (equal), methodology (equal), project administration (equal), resources (equal), supervision (equal), validation (equal), visualization (equal), writing – original draft (equal), writing – review and editing (equal). **Aradhana Sahu:** conceptualization (equal), funding acquisition (equal), methodology (equal), project administration (equal), resources (equal), supervision (equal), validation (equal), visualization (equal). **Nityanand Srivastava:** funding acquisition (equal), project administration (equal), resources (equal), supervision (equal), validation (equal). **Rohit Chaudhary:** formal analysis (equal), investigation (equal), methodology (equal), software (equal), writing – original draft (equal), writing – review and editing (equal). **Lahar Jhala:** data curation (equal), investigation (equal), writing – review and editing (equal). **Yashpal Zala:** data curation (equal), formal analysis (equal), investigation (equal), software (equal).

## Ethics Statement

All scientific research activities conducted as part of this study on Asiatic lions were undertaken following the requisite permission obtained from the Ministry of Environment, Forests, and Climate Change (MoEF&CC), Government of India (Letter No.: F. No. 1‐50/2018 WL) and Chief Wildlife Warden, Gujarat State, Gandhinagar (Letter No.: WLP/26B/781‐83/2019‐20). The procedures and protocols adhered to were in accordance with the Standard Operating Procedures as per the Management Plan for Gir Protected Areas by the Gujarat Forest Department, Government of Gujarat.

## Conflicts of Interest

The authors declare no conflicts of interest.

## Supporting information


**Figures S1–S13.** Land use land‐cover maps showing movement track, movement locations and core area usage (50% FK) of studied individuals.
**Table S1.** Details of radio‐collared lions along with their core areas (50% FK in km^2^) in the present study.

## Data Availability

All data generated or analyzed during this study period are included in this article.
