# Supplementary material for: Long‐Ranging Movements of Asiatic Lions: Implications for Conservation and Management in Gujarat, India
Source: Ecol Evol. 2025 Jul 16;15(7):e71811. doi: 10.1002/ece3.71811 (PMC12266744; doi:10.1002/ece3.71811)
Supplement: Supplementary file 1 — Figures S1–S13. Land use land‐cover maps showing movement track, movement locations and core area usage (50% FK) of studied individuals. Table S1. Details of radio‐collared lions along with their core areas (50% FK in km2) in the present study. [file ECE3-15-e71811-s001.docx]

**Supplementary Material**

**(Long-ranging Movements of Asiatic Lions: Implications for Conservation and Management in Gujarat, India.)**


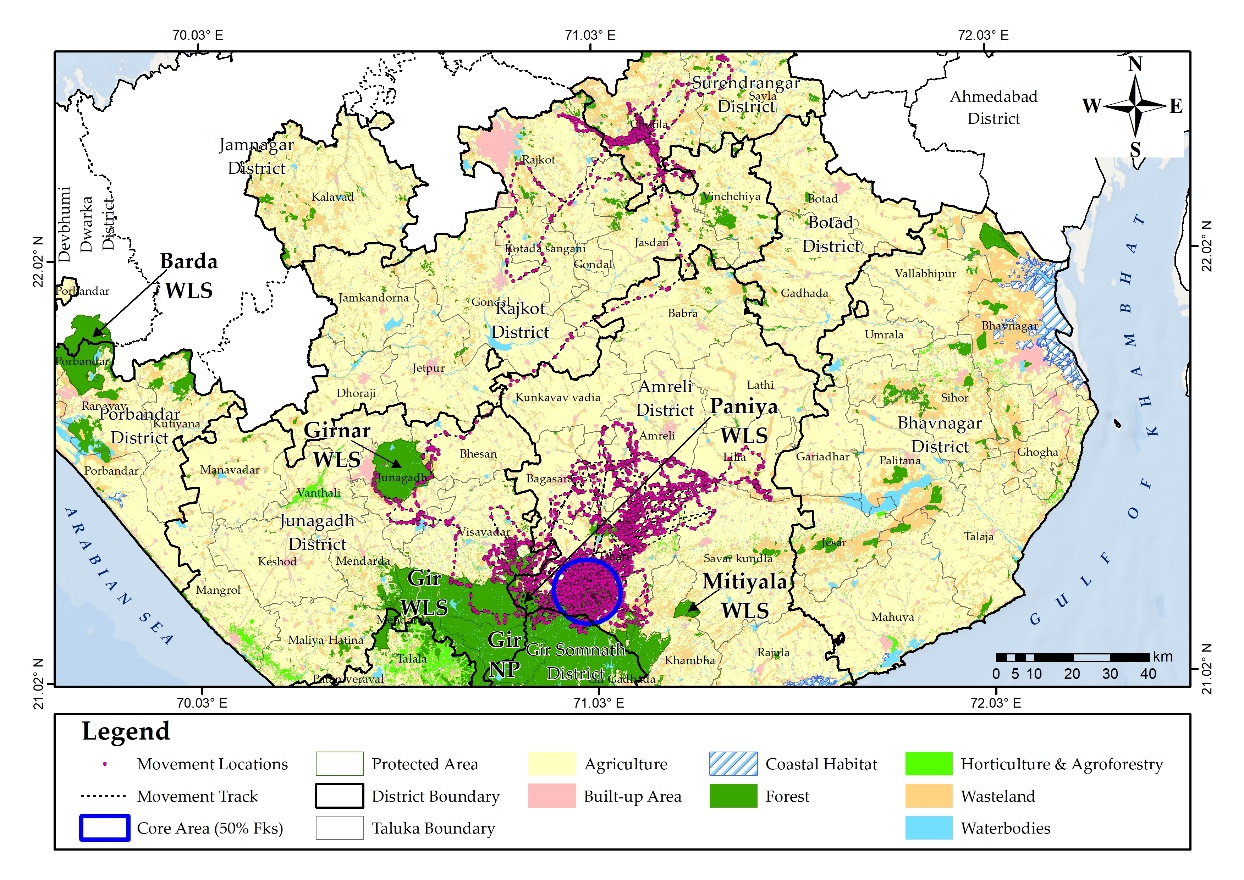


**Figure S1:** Land use land cover map showing movement track, movement locations and core area usage (50% FK) of Chotila male.


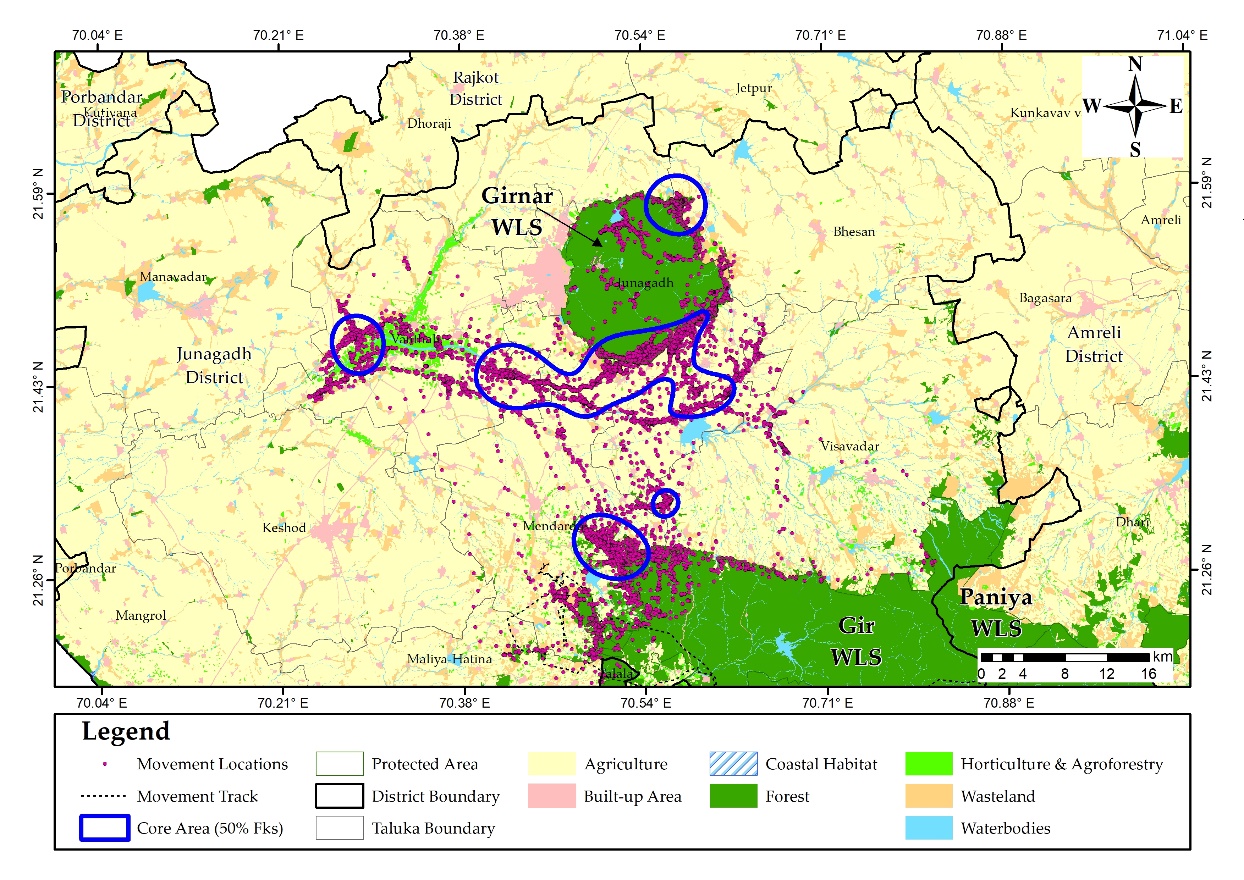


**Figure S2:** Land use land cover map showing movement track, movement locations and core area usage (50% FK) of Dedakadi male.


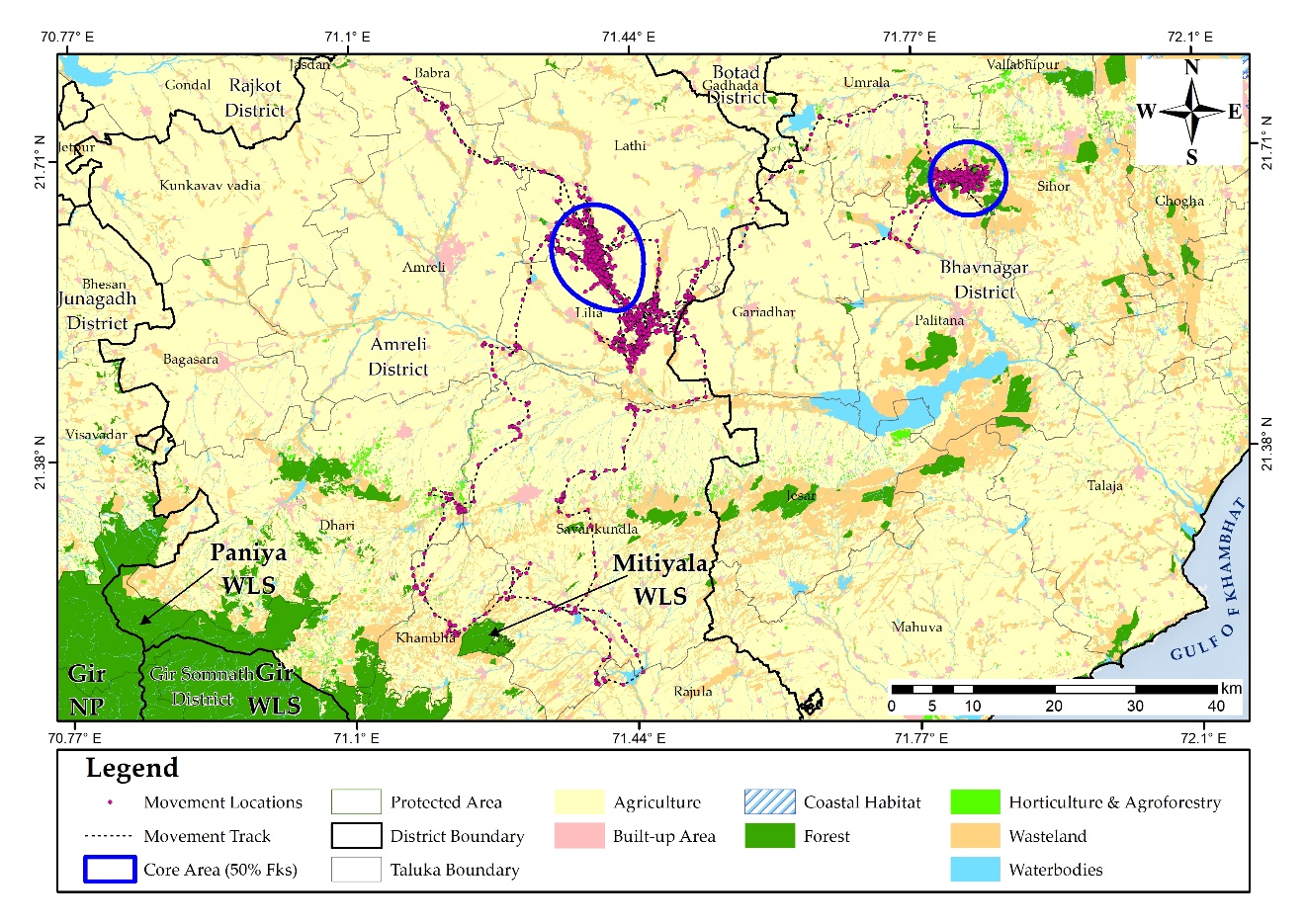


**Figure S3:** Land use land cover map showing movement track, movement locations and core area usage (50% FK) of Gariyadhar male.


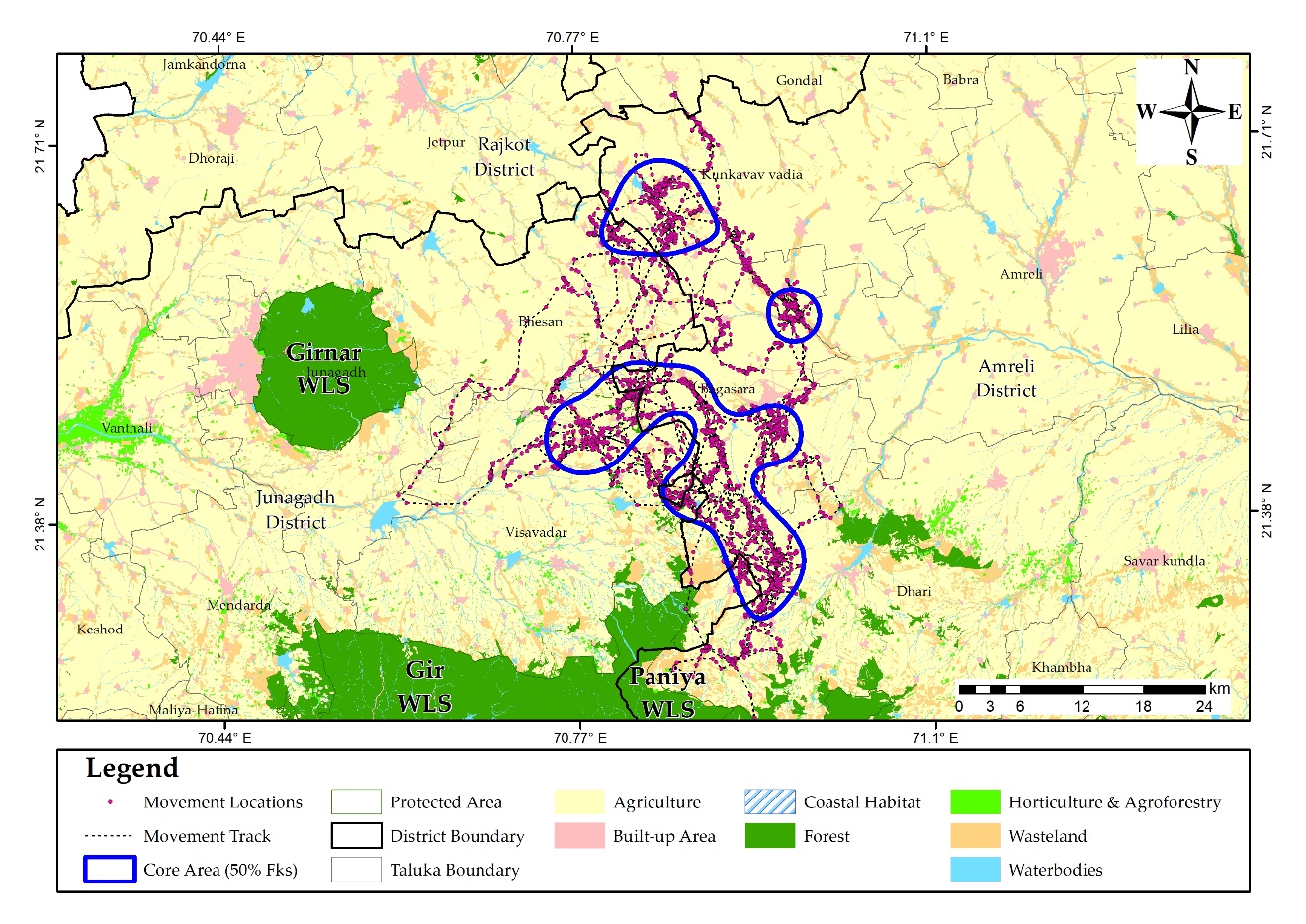


**Figure S4:** Land use land cover map showing movement track, movement locations and core area usage (50% FK) of Hadala female 1.


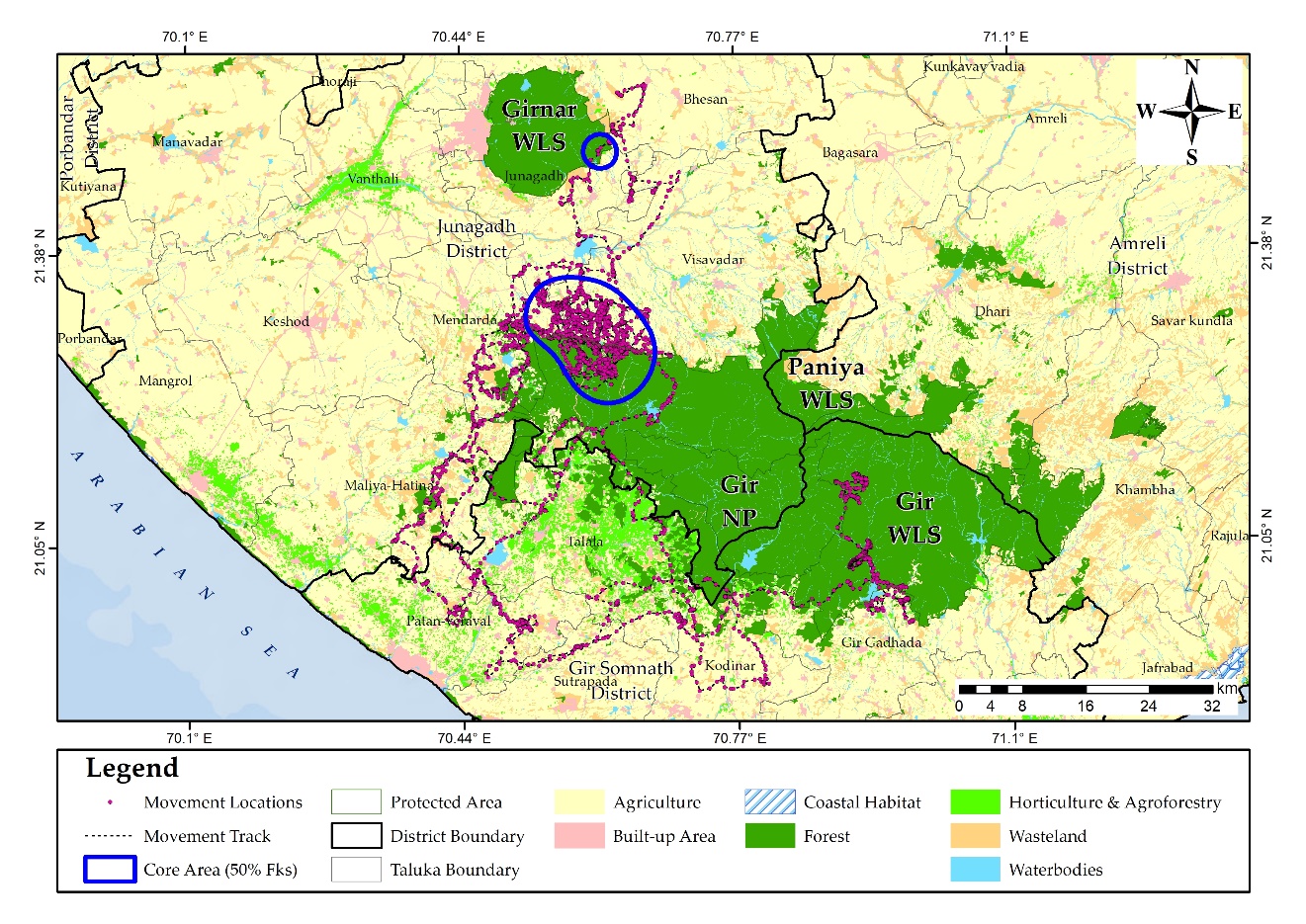


**Figure S5:** Land use land cover map showing movement track, movement locations and core area usage (50% FK) of Hadala female 2.


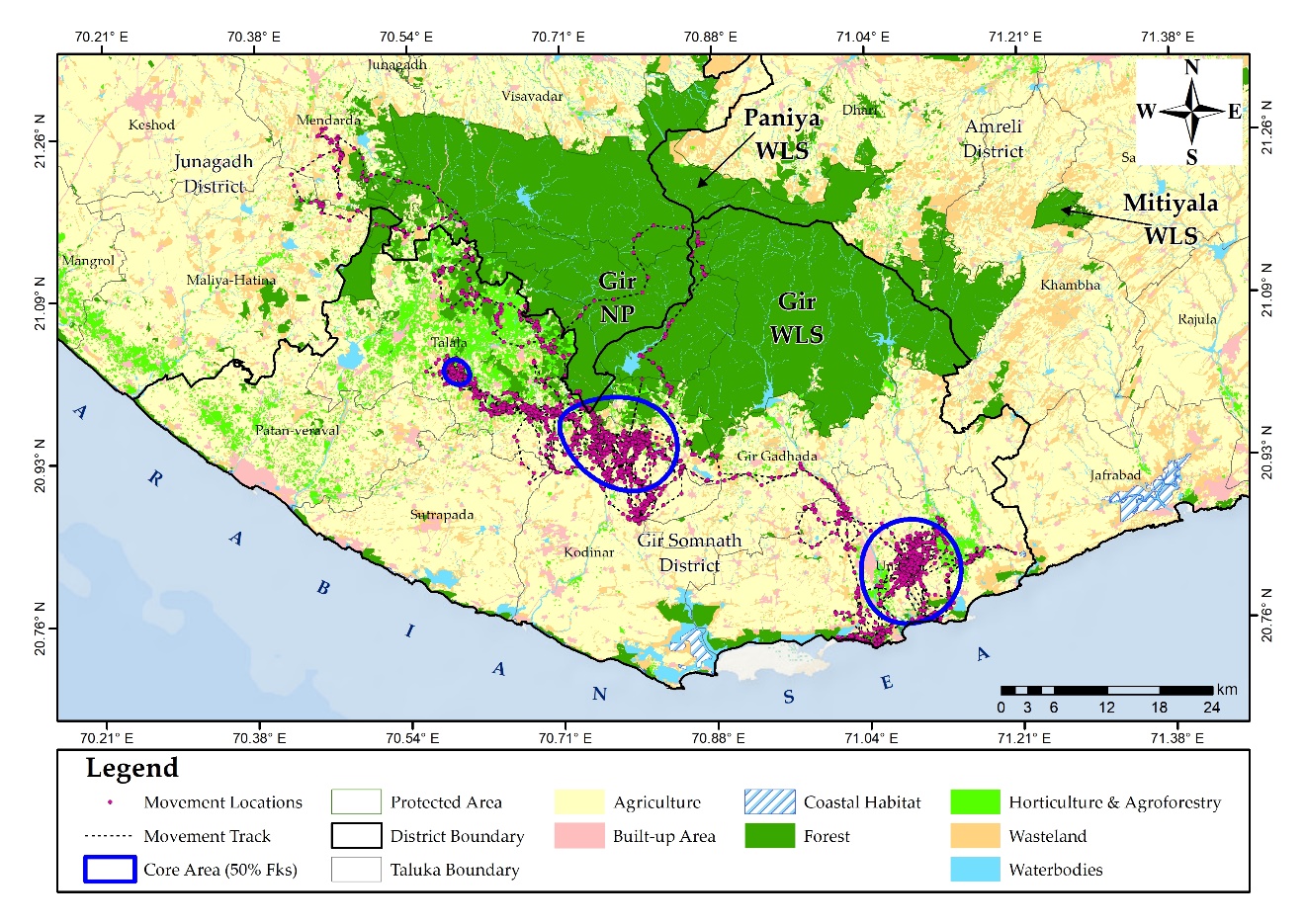


**Figure S6:** Land use land cover map showing movement track, movement locations and core area usage (50% FK) of Jasadhar male.


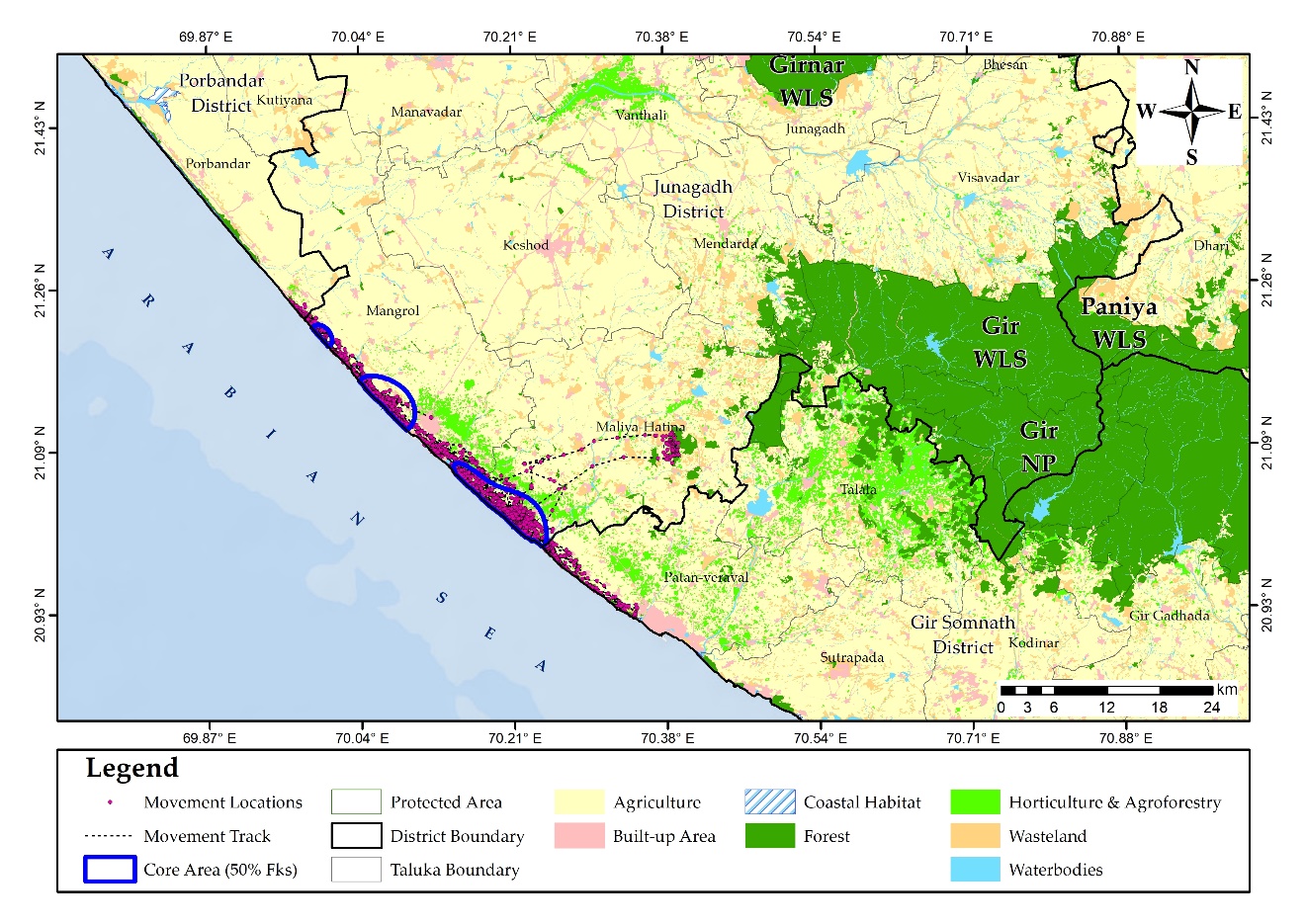


**Figure S7:** Land use land cover map showing movement track, movement locations and core area usage (50% FK) of Ranavav female.


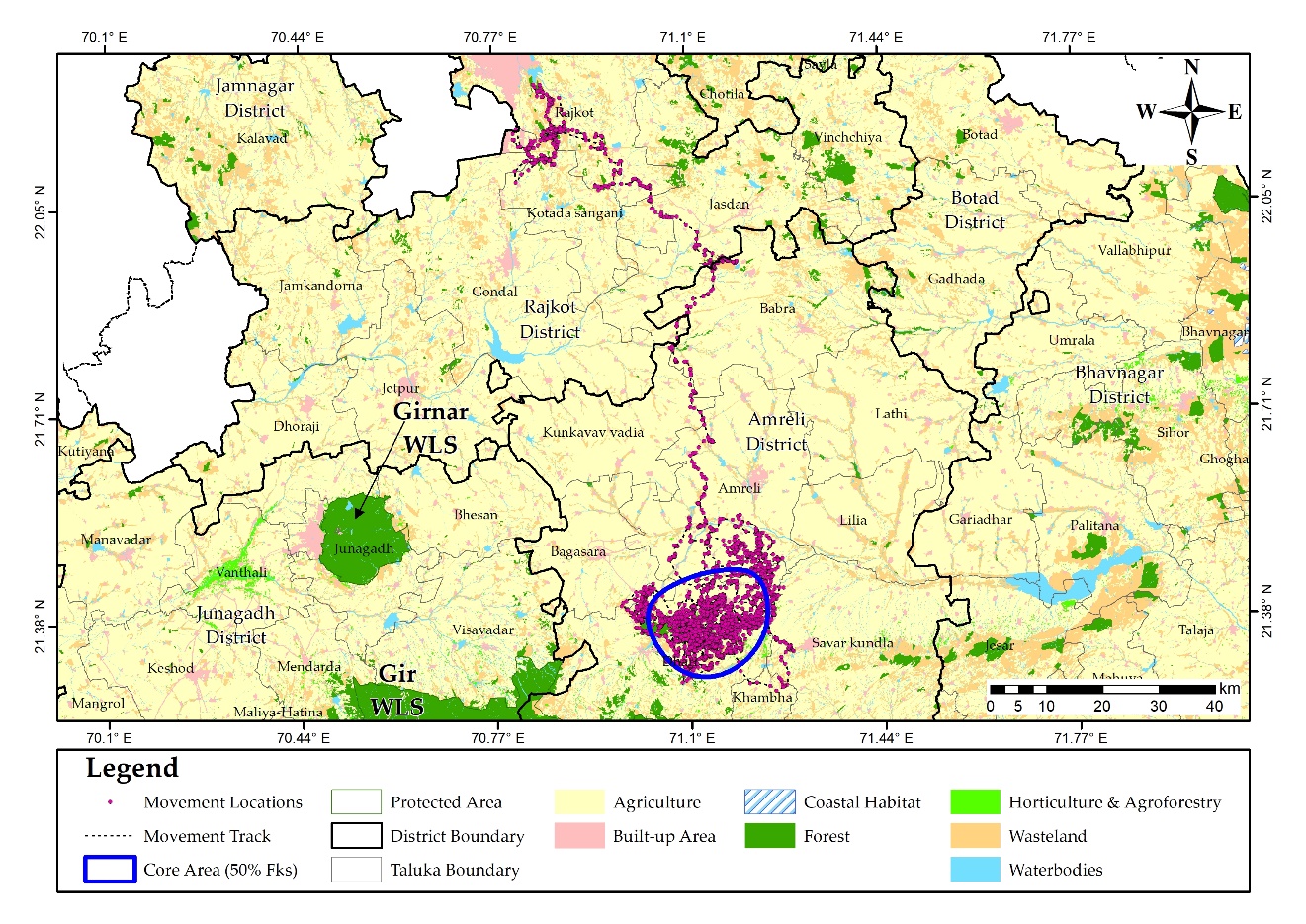


**Figure S8:** Land use land cover map showing movement track, movement locations and core area usage (50% FK) of Sarasiya male.


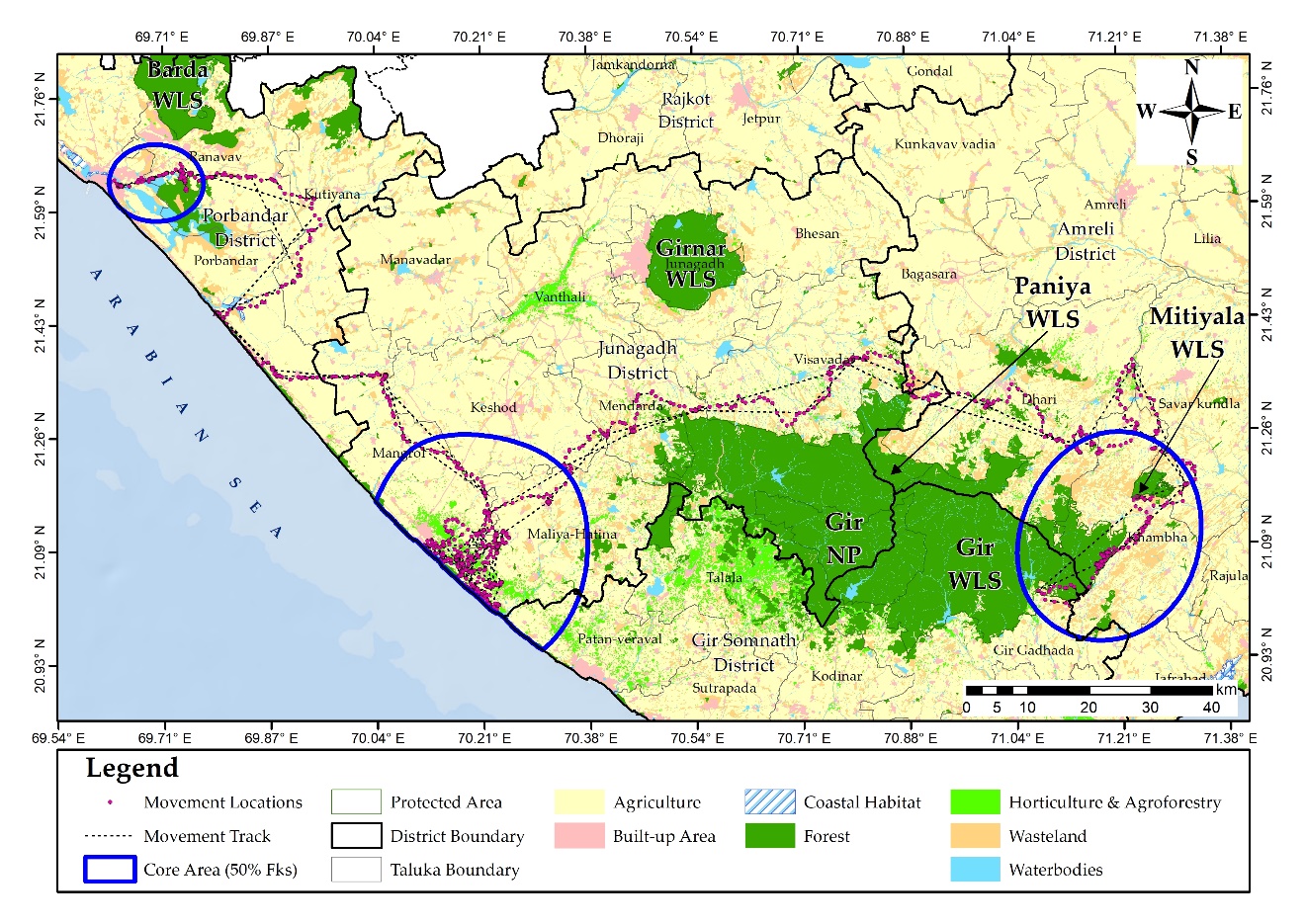


**Figure S9:** Land use land cover map showing movement track, movement locations and core area usage (50% FK) of Tulsishyam female.


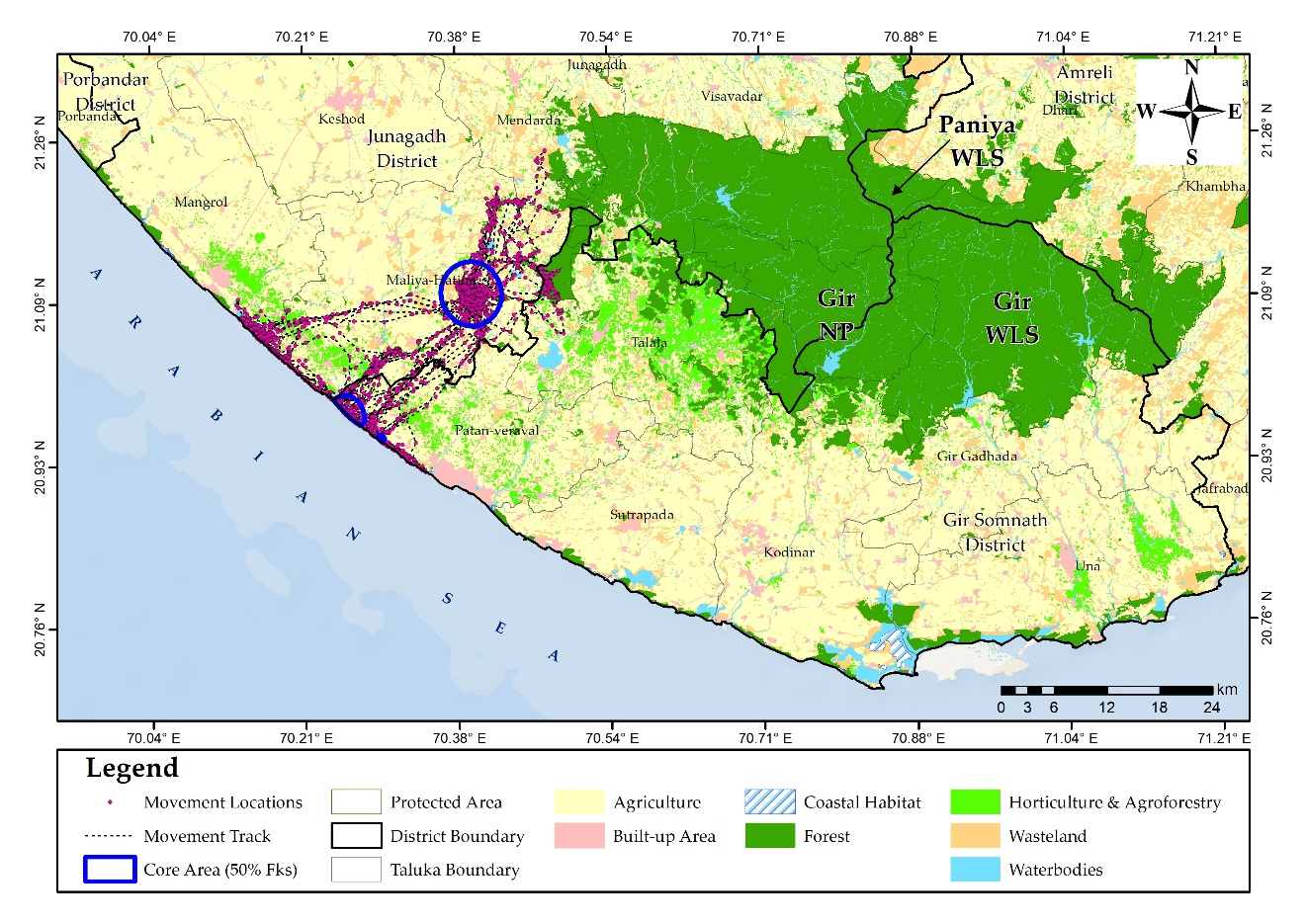


**Figure S10:** Land use land cover map showing movement track, movement locations and core area usage (50% FK) of Veraval male.

Table S1: Details of radio-collared lions along with their core areas (50% FK in km^2^) in the present study.

| **Sr. No.** | **Lion’s ID** | **Sex** | **Age**  **(Years)** | **Deployment Date** | **Data upto** | **No. of Locations** | **Group Composition** | | | | **Core Area (50% FK, km^2^)** |
| --- | --- | --- | --- | --- | --- | --- | --- | --- | --- | --- | --- |
|  |  |  |  |  |  |  | **Male** | **Female** | **Cub** | **Total** |  |
| 1 | Chotila Male | Male | 3-5 | 03-12-2019 | 04-11-2022 | 24309 | 2 | 0 | 0 | 2 | 373.40 |
| 2 | Dedakadi Male | Male | 5-9 | 27-03-2021 | 12-12-2023 | 30523 | 1 | 1 | 0 | 2 | 199.51 |
| 3 | Gariyadhar Male | Male | 2.5-3 | 05-07-2019 | 11-07-2020 | 8754 | 2 | 1 | 0 | 3 | 171.61 |
| 4 | Hadala Female - 1 | Female | 5-9 | 08-12-2022 | 12-12-2023 | 8672 | 0 | 2 | 6 | 8 | 355.15 |
| 5 | Hadala Female - 2 | Female | 8-10 | 19-01-2023 | 12-12-2023 | 15317 | 0 | 1 | 4 | 5 | 178.99 |
| 6 | Jasadhar Male | Male | 4-5 | 25-06-2019 | 07-10-2020 | 10605 | 1 | 2 | 0 | 3 | 200.15 |
| 7 | Ranavav Female | Female | 7-8 | 05-12-2020 | 12-10-2021 | 7177 | 0 | 1 | 2 | 3 | 90.27 |
| 8 | Sarasiya Male | Male | 2-3 | 06-01-2020 | 12-01-2021 | 8246 | 1 | 3 | 0 | 4 | 306.82 |
| 9 | Tulsishyam Female | Female | 5-9 | 16-07-2023 | 16-11-2023 | 5395 | 0 | 1 | 4 | 5 | 1921.95 |
| 10 | Veraval Male | Male | 3-5 | 22-06-2019 | 27-07-2020 | 9058 | 1 | 0 | 0 | 1 | 44.62 |
